# Repeatability of fully automated, inline quantitative assessment of myocardial perfusion in patients with suspected coronary artery disease

**DOI:** 10.1093/ehjimp/qyaf026

**Published:** 2025-03-05

**Authors:** Mohamed Elshibly, Charley Budgeon, Simran Shergill, Rachel England, Kelly Parke, Aida Moafi, Hui Xue, Peter Kellman, Gerry P McCann, Jayanth R Arnold

**Affiliations:** Department of Cardiovascular Sciences, University of Leicester, and the National Institute for Health and Care Research (NIHR) Leicester Cardiovascular Biomedical Research Centre, Glenfield Hospital, Leicester LE3 9QP, UK; Department of Cardiovascular Sciences, University of Leicester, and the National Institute for Health and Care Research (NIHR) Leicester Cardiovascular Biomedical Research Centre, Glenfield Hospital, Leicester LE3 9QP, UK; Cardiovascular Epidemiology Research Centre, University of Western Australia, Perth, Australia; Department of Cardiovascular Sciences, University of Leicester, and the National Institute for Health and Care Research (NIHR) Leicester Cardiovascular Biomedical Research Centre, Glenfield Hospital, Leicester LE3 9QP, UK; Department of Cardiovascular Sciences, University of Leicester, and the National Institute for Health and Care Research (NIHR) Leicester Cardiovascular Biomedical Research Centre, Glenfield Hospital, Leicester LE3 9QP, UK; Department of Cardiovascular Sciences, University of Leicester, and the National Institute for Health and Care Research (NIHR) Leicester Cardiovascular Biomedical Research Centre, Glenfield Hospital, Leicester LE3 9QP, UK; Department of Cardiovascular Sciences, University of Leicester, and the National Institute for Health and Care Research (NIHR) Leicester Cardiovascular Biomedical Research Centre, Glenfield Hospital, Leicester LE3 9QP, UK; National Heart, Lung, and Blood Institute, National Institutes of Health, Bethesda, MD, USA; National Heart, Lung, and Blood Institute, National Institutes of Health, Bethesda, MD, USA; Department of Cardiovascular Sciences, University of Leicester, and the National Institute for Health and Care Research (NIHR) Leicester Cardiovascular Biomedical Research Centre, Glenfield Hospital, Leicester LE3 9QP, UK; Department of Cardiovascular Sciences, University of Leicester, and the National Institute for Health and Care Research (NIHR) Leicester Cardiovascular Biomedical Research Centre, Glenfield Hospital, Leicester LE3 9QP, UK

**Keywords:** cardiovascular magnetic resonance, quantitative perfusion, repeatability, stable coronary artery disease

## Abstract

**Aims:**

Recent developments in the field of myocardial perfusion assessment with cardiovascular magnetic resonance (CMR) enable the automated inline quantification of myocardial blood flow (MBF). Previous studies have assessed its repeatability in healthy volunteers. This study assessed the repeatability of this technique in patients with suspected stable coronary artery disease (CAD).

**Methods and results:**

Patients with suspected CAD were studied twice on separate days. CMR perfusion imaging was performed at rest and during adenosine stress using a dual-sequence T1-weighted saturation recovery gradient echo sequence. Inline automatic reconstruction and image post-processing were implemented within the Gadgetron software framework, calculating MBF using a blood tissue exchange model. Repeatability of global stress and rest MBF, and myocardial perfusion reserve (MPR) were evaluated using Bland-Altman plots and intraclass correlation coefficients. Fifty-four patients (mean age 67 ± 9 years, 78% male) were studied. The median interval between the two scans was 2 days (IQR 3). There was no significant interstudy difference in global stress MBF (1.46 ± 0.51 mL/min/g vs. 1.51 ± 0.59mLmin/g, *P* = 0.44), global rest MBF (0.54 ± 0.14 mL/min/g vs. 0.56 ± 0.16 mL/min/g, *P* = 0.48), or global MPR (2.72 ± 0.80 vs. 2.84 ± 1.13, *P* = 0.76) between the two scans. Stress MBF, rest MBF, and MPR showed intraclass correlations of 0.60 (95% CI 0.39–0.75), 0.63 (95% CI 0.36–0.77), and 0.39 (95% CI 0.09–0.62), respectively.

**Conclusion:**

In patients with suspected CAD, quantitative assessment of myocardial perfusion by fully automated inline myocardial mapping shows moderate repeatability for stress and rest MBF, but poorer repeatability with MPR.

## Graphical Abstract

**Graphical Abstract:**
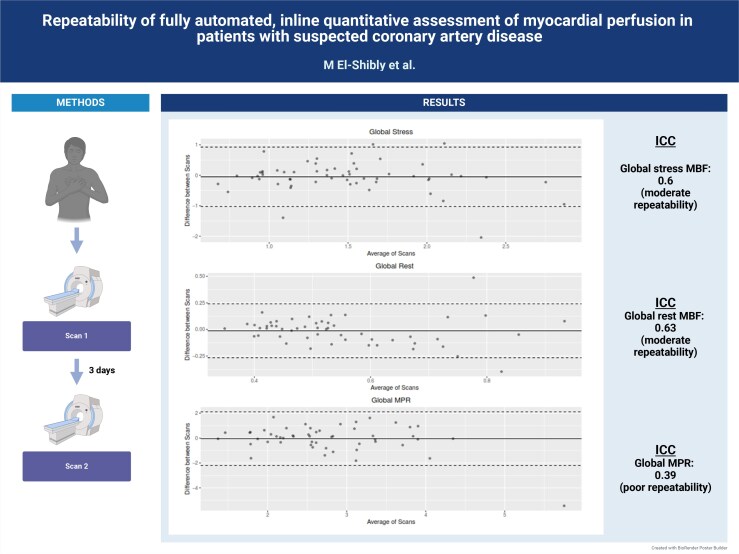

## Introduction

Myocardial perfusion imaging with cardiovascular magnetic resonance (CMR) is an established clinical imaging modality affording valuable diagnostic and prognostic information in patients with suspected coronary artery disease (CAD).^1^ Beyond qualitative, visual assessment of images, absolute quantification of myocardial blood flow (MBF) may offer superior discrimination of the severity and extent of ischaemia, notably for the detection of multi-vessel and microvascular CAD.^2^

Recent developments in this field include a novel perfusion sequence involving automated inline quantitative assessment of myocardial perfusion,^3^ with validation against positron emission tomography.^4^ Visual assessment of perfusion is subjective and may underestimate the extent and severity of ischaemia compared with quantitative assessment. The latter affords objectivity and superior diagnostic discrimination of multi-vessel CAD^2^ and incremental prognostic information beyond visual analysis alone.^5^ A quantitative perfusion approach may also offer clinical utility in reducing variability between studies and ensure reliable monitoring of disease progression, which may play a role in patients with CAD to assess for serial changes following therapeutic interventions such as revascularisation.^6^ Given the potential utility of quantitative CMR perfusion imaging in repeated studies of myocardial perfusion, it is vital that this technique yields reproducible results. Previous work assessing the repeatability of inline quantitative CMR myocardial perfusion assessment has been limited to healthy subjects, thus underscoring the need to examine repeatability in diverse patient cohorts.^7^ This includes patients with CAD, in whom regional coronary flow heterogeneities may be associated with lower repeatability.

In this prospective clinical study, we sought to compare the repeatability of quantitative CMR perfusion in patients with suspected CAD.

## Methods

Patients were recruited prospectively from a single tertiary cardiac centre (Glenfield Hospital, Leicester, United Kingdom). The study involved patients with suspected angina referred for clinically indicated invasive coronary angiography (ICA). Prior to this, subjects underwent two CMR perfusion scans on separate days up to one week apart. Exclusion criteria were acute coronary syndromes, second/third-degree atrioventricular block, severe chronic obstructive pulmonary disease, moderate-severe asthma, estimated glomerular filtration rate <30 mL/min/1.73 m^2^, severe claustrophobia, and absolute contraindications to CMR. The study was approved by the regional institutional ethics committee (19/EM/0295). It was conducted according to the principles of Good Clinical Practice and according to the Declaration of Helsinki, under the oversight of the University of Leicester.

### Study protocol

All CMR scans were performed at 3-Tesla (Magnetom Vida or Skyra, Siemens Healthineers, Erlangen, Germany). Each participant underwent both scans in the same scanner. They were advised to abstain from caffeine for 12 hours prior to each scan. Following standard pilot and localizer images, functional cine images were acquired according to standard clinical protocols using a steady-state free precession pulse sequence in the three long-axis views and a contiguous short-axis stack to cover the ventricles. Typical parameters were slice thickness 8 mm, distance factor 25%, matrix 256 × 204, field of view variable 300–360 × 360–420 mm, TR 45 ms and flip angle 45°. Using the long-axis images for planning, perfusion assessment was performed in three short-axis planes to cover the basal, mid-ventricular and apical levels of the left ventricle, using a dual-sequence T1-weighted saturation recovery gradient echo sequence acquired over 60 heartbeats, with intravenous injection of 0.075 mmol/kg of a gadolinium-based contrast agent (Dotarem, Guerbet, France) followed by 20 mL saline bolus (injection speed 4 mL/s) for each scan. Typical parameters were a matrix of 224 × 134, slice thickness 8 mm, TR 176 ms, TE 1 ms and flip angle 10°. Inline automatic reconstruction and image post-processing were implemented within the Gadgetron software framework, with images corrected for motion and surface coil intensity variation and calculation of MBF using a blood tissue exchange model, displayed on pixel-wise perfusion maps as previously reported.^3,8^ Perfusion assessment was performed at rest and during pharmacological stress with intravenous infusion of adenosine beginning at a rate of 140 mcg/kg/min for a duration of 3–5 minutes. Subjects were monitored for symptoms throughout the infusion, and the dose was increased at 2-minute intervals to 170–210 mcg/kg/min if an insufficient haemodynamic response was achieved (heart rate increase ≥10 beats per minute).

### Quantitative analysis

Perfusion maps were blindly analysed offline using CVi42 software (v5.10.1 Circle Cardiovascular Imaging, Calgary, Canada). This was performed by manually tracing endocardial and epicardial contours for the basal, mid-ventricular and apical slices, and applying a 10% offset for each border. Segmental MBF was calculated, applying the 16-segment American Heart Association model.^9^ Myocardial perfusion reserve (MPR) was defined as the ratio of segmental hyperaemic MBF/rest MBF. Segments were grouped into coronary territories and assigned to either left anterior descending (LAD) artery, left circumflex artery (LCx) or right coronary artery (RCA) as previously described^9^ and adjusted for coronary dominance as identified during ICA (see Supplementary data online, *Table S1*).

### Statistical analysis

Continuous data are expressed as mean (±SD) or median (interquartile range), and scans are compared with paired *t*-tests. Categorical data are presented as number (percentage) of patients. The repeatability of stress and rest MBF, and MPR (global, regional and segmental measures) was assessed using intraclass correlation coefficient (ICC) and 95% confidence intervals (CI) obtained using bootstrapping. Agreement was visualised using Bland-Altman plots.^10^

## Results

Fifty-nine participants were recruited (78% male, mean age 67 ± 9 years). The median interval between studies was 2 days (IQR 3). Baseline characteristics are presented in *Table 1*. Hypertension, hypercholesterolaemia, smoking history, and the use of statins and aspirin were prevalent among the study participants. All subjects attended both visits and tolerated stress scans well. There were four failed automatic inline reconstruction of raw data (two stress perfusion and two rest perfusion scans) due to software malfunction, and one failed reconstruction of rest perfusion images, leaving 54 complete studies with paired data.

**Table 1:** Baseline characteristics

Demographics	
Age	67 ± 9
Male	39 (78%)
Never smoked	26 (52%)
Body mass index (kg/m^2^)	29.7 ± 3.9
Medical history	
Diabetes	7 (14%)
Hypertension	29 (58%)
Hypercholesterolaemia	23 (46%)
ACEi/ARB	18 (36%)
Beta blocker	28 (56%)
Calcium channel blocker	14 (28%)
Statin	43 (86%)
Aspirin	42 (84%)
Anticoagulant	2 (4%)
CMR	
LVEF (%)	60.1 ± 9.7
LVEDVi (mL/m^2^)	77.8 ± 16.5
LVESVi (mL/m^2^)	31.4 ± 11.0
LVMi (g/m^2^)	62.1 ± 12.7

*n* (%) or mean ± SD.

### Haemodynamic data

*Table 2* summarises the haemodynamic response to adenosine. There were no significant interstudy differences in resting and peak stress heart rate or systolic blood pressure.

**Table 2:** Haemodynamic response to adenosine infusion

	Scan 1	Scan 2	*P*-value
Resting heart rate, bpm	61 ± 11	62 ± 12	0.068
Peak stress heart rate, bpm	81 ± 13	82 ± 13	0.404
Resting systolic blood pressure, mmHg	135 ± 22	138 ± 21	0.159
Stress systolic blood pressure, mmHg	133 ± 21	130 ± 18	0.059

Mean ± SD

### Global and regional (segmental) perfusion analysis

**Figure 1:**
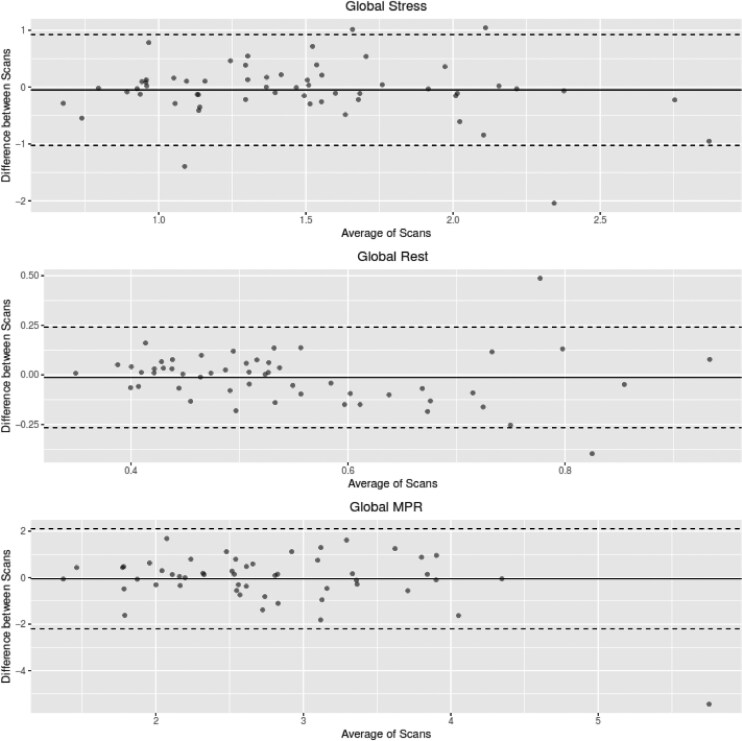
Bland-Altman plots for global stress MBF, global rest MBF and MPR.

**Figure 2:**
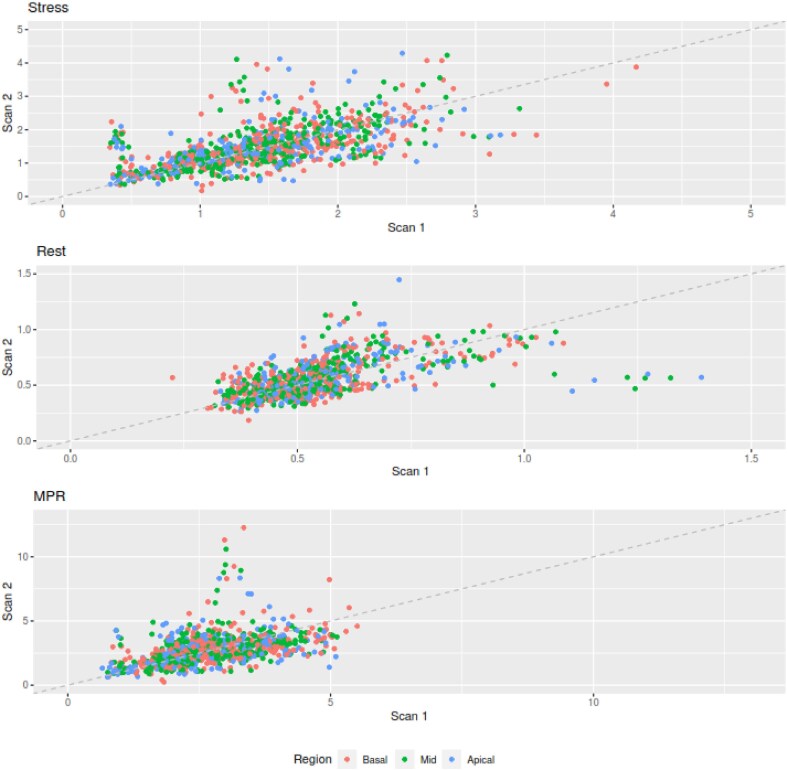
Scatter plots for stress MBF, rest MBF and MPR across basal (red), mid (green) and apical (blue) segments.

Fifty-four studies were analysed for interstudy repeatability of global stress MBF, rest MBF, and MPR. There was no significant difference in global stress MBF between the two scans (1.46 ± 0.51 mL/min/g vs. 1.51 ± 0.59 mLmin/g, *P* = 0.44), global rest MBF (0.54 ± 0.16 mL/min/g vs. 0.56 ± 0.16 mL/min/g, *P* = 0.48) or global MPR (2.72 ± 0.80 vs. 2.84 ± 1.13, *P* = 0.76). Stress MBF, rest MBF, and MPR showed intraclass correlations of 0.60 (95% CI 0.39–0.75), 0.63 (95% CI 0.36–0.77), and 0.39 (95% CI 0.09–0.62), respectively. (*Table 3* and *Figure 1*). In per-segment analysis, interstudy repeatability demonstrated comparable results, with moderate reproducibility across all measures. (*Table 4* and *Figure 2*).

**Table 3:** Mean global stress MBF, global rest MBF and MPR, and ICC between scans

	Scan 1	Scan 2	*P*-value	ICC (95% CI)
Global Stress MBF (mL/min/g)	1.46 ± 0.51	1.51 ± 0.59	0.442	0.60 (0.39, 0.75)
Global Rest MBF (mL/min/g)	0.54 ± 0.14	0.56 ± 0.16	0.479	0.63 (0.36, 0.77)
Global MPR	2.72 ± 0.80	2.84 ± 1.13	0.760	0.39 (0.09, 0.62)

Mean ± SD.

**Table 4:** Regional (segmental) stress and rest MBF, and MPR, and ICC between scans

Slice (segments)	Scan 1	Scan 2	*P*-value	ICC
Basal (1–6)				
Stress	1.49 ± 0.53	1.55 ± 0.60	0.474	0.60 (0.39, 0.78)
Rest	0.54 ± 0.13	0.56 ± 0.16	0.296	0.68 (0.52, 0.82)
MPR	2.80 ± 0.83	2.92 ± 1.17	0.791	0.40 (0.08, 0.65)
Mid (7–12)				
Stress	1.43 ± 0.50	1.49 ± 0.59	0.414	0.59 (0.26, 0.75)
Rest	0.54 ± 0.15	0.56 ± 0.16	0.652	0.60 (0.37, 0.77)
MPR	2.68 ± 0.80	2.80 ± 1.13	0.727	0.37 (0.04, 0.63)
Apical (13–16)				
Stress	1.44 ± 0.55	1.50 ± 0.62	0.441	0.59 (0.33, 0.78)
Rest	0.55 ± 0.15	0.57 ± 0.16	0.577	0.53 (0.23, 0.74)
MPR	2.66 ± 0.90	2.78 ± 1.15	0.742	0.40 (0.12, 0.68)

Mean ± SD.

### Regional perfusion analysis by coronary artery territory

**Figure 3:**
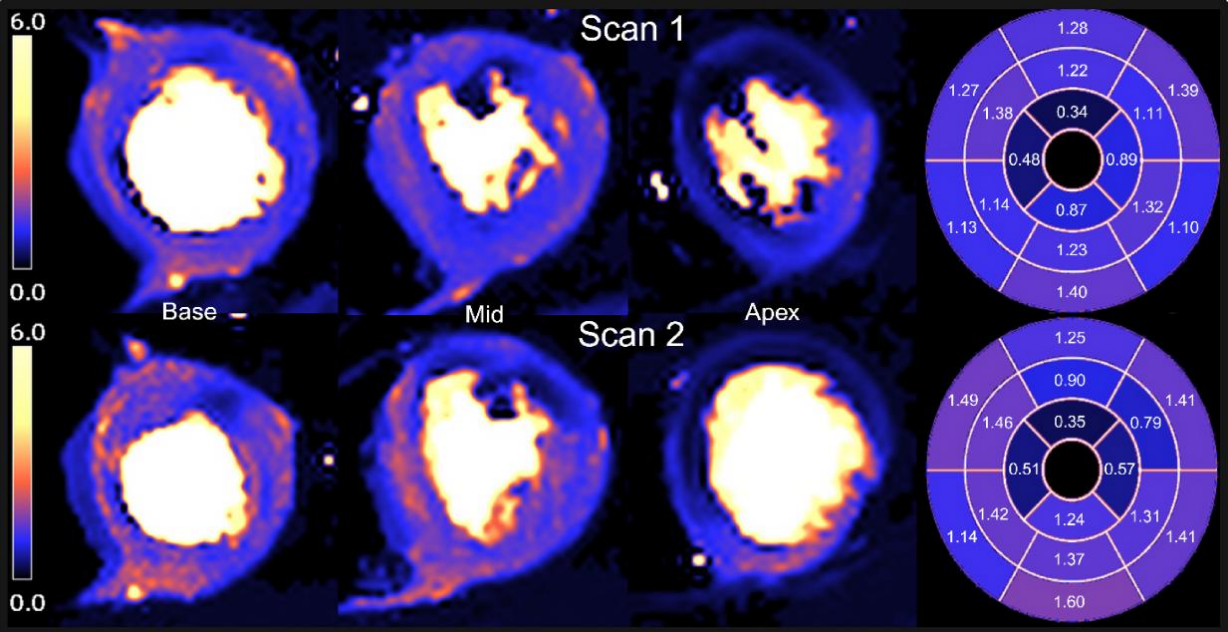
Pixel-wise hyperaemic perfusion maps for Scan 1 and Scan 2 show a defect in the LAD and LCx artery territories with corresponding regional reductions in hyperaemic MBF in the affected segments on polar plot maps.

There was no significant interstudy difference in stress MBF, rest MBF, and MPR in each of the three coronary artery territories. ICC’s revealed moderate repeatability for stress and rest MBF, and poorer repeatability for MPR, between both scans. (*Table 5* and *Figure 3*).

**Table 5:** Mean coronary artery territory stress MBF, rest MBF and MPR

	Scan 1	Scan 2	*P*-value	ICC
Stress				
LAD	1.52 ± 0.55	1.57 ± 0.66	0.606	0.59 (0.37, 0.78)
LCx	1.50 ± 0.55	1.57 ± 0.62	0.536	0.61 (0.26, 0.79)
RCA	1.31 ± 0.49	1.38 ± 0.54	0.311	0.62 (0.30, 0.77)
Rest				
LAD	0.56 ± 0.14	0.58 ± 0.17	0.484	0.56 (0.26, 0.78)
LCx	0.54 ± 0.14	0.55 ± 0.16	0.736	0.69 (0.44, 0.85)
RCA	0.51 ± 0.12	0.53 ± 0.15	0.298	0.63 (0.32, 0.79)
MPR				
LAD	2.75 ± 0.87	2.83 ± 1.13	0.909	0.44 (0.08, 0.71)
LCx	2.82 ± 0.84	2.98 ± 1.35	0.712	0.37 (0.12, 0.62)
RCA	2.57 ± 0.86	2.71 ± 1.06	0.577	0.43 (0.12, 0.69)

Mean ± SD.

## Discussion

To our knowledge, this is the first study to assess the repeatability of CMR quantitative perfusion assessment in patients with suspected CAD. We have shown that this technique has moderate repeatability for stress and rest MBF, but poorer repeatability for MPR. These results have implications for determining appropriate sample sizes in future studies and underscore the importance of exercising caution when interpreting serial changes in quantitative parameters.

Quantitative perfusion assessment with CMR affords superior accuracy and objectivity compared with standard qualitative, visual assessment.^2^ A prospective study involving 151 patients undergoing ICA with fractional flow reserve measurement showed that, compared with standard qualitative assessment, quantitative perfusion mapping identified greater ischaemic burden and hence, is better able to detect the extent of CAD.^2^ In another study of 50 patients with stable CAD undergoing ICA with invasive coronary physiology, quantitative CMR perfusion assessment accurately discriminated between epicardial CAD and microvascular dysfunction.^11^ Furthermore, the prognostic capability of quantitative perfusion assessment has been shown in a study involving 1049 patients (median follow-up 600 days), demonstrating that reduced hyperaemic MBF and MPR are strong independent predictors of major adverse cardiac events.^5^

The objective nature of quantitative perfusion assessment makes this a potentially valuable tool for serial clinical evaluation in different patient populations, in monitoring disease progression, and in evaluating the efficacy of clinical interventions (pharmacotherapy and/or coronary intervention). In addition to clinical applications, serial, quantitative assessment of perfusion may serve as a useful surrogate endpoint in clinical trials.^6^ However, for appropriate precision in serial imaging studies, it is critical that this technique yields reproducible results.

In the literature, there are relatively few studies assessing the reproducibility of quantitative CMR perfusion, mostly involving small numbers of predominantly healthy participants. In a study of 16 subjects (7 normal volunteers and 9 CAD patients, with a mean interstudy interval of 13 ± 8 days), a Fermi deconvolution approach demonstrated good reproducibility. The global MPR coefficient of variation (CV), defined as the standard deviation of the measurement differences divided by the mean, was 21% in volunteers and 23% in CAD patients.^12^ Another study involving two scans on the same day in 11 healthy volunteers showed moderate reproducibility (CV 16.0, 26.8, and 23.9%) for global rest and stress perfusion and MPR, with no significant diurnal variation.^13^ Jerosch-Herold *et al.*^14^ reported reproducibility coefficients of 30 and 40% for rest and stress MBF, respectively, in a subgroup of the Multi-Ethnic Study of Atherosclerosis study, involving 30 subjects studied nearly a year between scans.^14^ Of note, in these studies, there are differences in field-strength, imaging sequences, and importantly, perfusion models for absolute quantification of perfusion.

More recently, Brown *et al.*^7^ using the same perfusion sequence and quantification model as utilised in the present study, assessed repeatability in 42 healthy subjects studied with a mean interstudy interval of 41 ± 40 days.^7^ This study demonstrated moderate interstudy repeatability for global stress MBF and rest MBF, and poor repeatability for MPR (ICC 0.72, 0.74, and 0.46, respectively). Furthermore, regional perfusion analysis revealed moderate repeatability of stress and rest MBF in all coronary artery territories (ICC 0.66–0.75), and poorer repeatability of MPR (ICC 0.23–0.54).

Consistent with this earlier study involving healthy volunteers, our study, involving patients with suspected CAD, demonstrated moderate interstudy repeatability for global stress MBF and rest MBF, and poorer repeatability of MPR (ICC 0.60, 0.63, and 0.39, respectively). Similarly, for regional assessment, it demonstrated moderate repeatability for stress MBF and rest MBF (ICC 0.53–0.68), and poorer repeatability for MPR (ICC 0.37–0.40). In keeping with the published literature, rest perfusion appears to be more reproducible than stress, and stress more reproducible than MPR. The lower repeatability of MPR may stem in part from its derivation process, whereby small differences in resting or hyperaemic flow can amplify variability in MPR. Rest MBF may be influenced by physiological variations between studies, whilst stress MBF may be affected by sampling different slice locations. These differences may be compounded by the regional coronary flow heterogeneities that occur in the presence of CAD.^15^ While an ICC value above 0.75 would be desirable to inform clinical decision-making, this threshold was not met consistently in our study, particularly in the context of MPR. The variability in MPR highlights the need for larger-scale studies across diverse patient populations prior to establishing the clinical utility of interpreting serial changes in perfusion.

The slightly lower repeatability of stress and rest MBF in our study compared with that of Brown *et al.*^7^ may relate to greater intrinsic variability of regional myocardial perfusion in our cohort, by virtue of their older age, comorbidities, higher use of prescription medications, and the presence of CAD. Other potential confounders include dietary factors, which have been shown to impact endothelial function^16^ and variable caffeine intake.^17^ Although we anticipated that subjects might be more anxious at their first visit, we identified no difference in resting heart rate or haemodynamic responses with adenosine.

## Limitations

This prospective study is the first to assess the repeatability of quantitative perfusion in a clinically relevant population of patients with suspected CAD, extending beyond previous studies in healthy volunteers. However, this single-centre study with a relatively modest sample size necessitates further validation in larger cohorts before broader clinical recommendations can be made. Furthermore, potential variabilities may arise in different perfusion slice prescriptions sampled by different radiographers conducting the two scans, diurnal variations in perfusion, and differences in the duration and peak dose of adenosine received between scans or a prolonged hyperaemic effect from adenosine leading to an overestimation of rest MBF.^18^ This could be addressed in future studies by standardising scan times, minimising operator variability with matched slice prescriptions, and using fixed adenosine infusion protocols with an appropriate offset prior to rest imaging. Dietary factors including caffeine intake were not strictly controlled, relying on participant compliance and may have affected the vasodilator response to adenosine. Previous studies have demonstrated that up to 20% of patients have detectable levels of caffeine despite reported abstention.^19^ Future research with larger-scale datasets in varying clinical populations, including model-, vendor-, and field-strength specific data is necessary prior to routine clinical implementation of an automated quantitative workflow.

## Conclusions

In patients with suspected CAD, fully automated, inline quantitative assessment of myocardial perfusion by myocardial mapping shows moderate reliability for stress and rest MBF, and poor reliability with MPR.

## Supplementary Material

qyaf026_Supplementary_Data

## Data Availability

The data underlying this article will be shared on reasonable request to the corresponding author.

## Lead author biography

Dr Mohamed Elshibly is a UK-based specialist registrar in cardiology with a focus on cardiovascular research. Dr Elshibly is currently working towards obtaining a PhD from the University of Leicester and has focused his career on advancing the understanding of myocardial perfusion and CAD. With expertize in CMR imaging, Dr Elshibly has contributed to key research projects aimed at improving diagnostic techniques and patient care. He is actively involved in clinical trials and academic collaborations, working to translate research findings into practical applications that benefit patients with cardiovascular conditions.
