# Supplementary material for: Repeatability of fully automated, inline quantitative assessment of myocardial perfusion in patients with suspected coronary artery disease
Source: Eur Heart J Imaging Methods Pract. 2025 Mar 5;2(4):qyaf026. doi: 10.1093/ehjimp/qyaf026 (PMC11935528; doi:10.1093/ehjimp/qyaf026)
Supplement: qyaf026_Supplementary_Data [file qyaf026_supplementary_data.docx]

**Supplementary table 1:** Myocardial segments with corresponding coronary territory

| **Segments** | **LAD** | **LCx** | **RCA** |
| --- | --- | --- | --- |
| Right dominant circulation | 1, 2, 7, 8, 13, 14 | 5, 6, 11, 12, 16 | 3, 4, 9, 10, 15 |
| Left dominant circulation | 1, 2, 7, 8, 13, 14 | 4, 5, 6, 10, 11, 12, 15, 16 | 3, 9 |
